# Expanding the phenotype associated with *SMARCC2* variants: a fetus with tetralogy of Fallot

**DOI:** 10.1186/s12920-022-01185-0

**Published:** 2022-03-03

**Authors:** Hairui Sun, Siyao Zhang, Jingyi Wang, Xiaoxue Zhou, Hongjia Zhang, Huixia Yang, Yihua He

**Affiliations:** 1 Department of Echocardiography, Beijing Anzhen Hospital, Capital Medical University, No.2, Anzhen Road, Chaoyang District, Beijing, 100029 China; 2 Department of Cardiac Surgery, Beijing Anzhen Hospital, Capital Medical University, No.2, Anzhen Road, Chaoyang District, Beijing, 100029 China; 3 Peking University First Hospital, Beijing, China

## Abstract

**Background:**

Coffin-Siris syndrome-8 (CSS8) is a rare autosomal dominant disorder caused by variants in *SMARCC2*, a core subunit of the chromatin-remodeling complex BRG1-associated factor (BAF). The clinical characteristics of this disorder have not been entirely determined because of the rarity of clinical reports. The BAF complex plays a crucial role in embryogenesis and cardiac development, and pathogenic variants in genes encoding the components of the BAF complex have been associated with congenital heart disease (CHD). However, variants in *SMARCC2* have not been reported in patients with CHD.

**Case presentation:**

A 28-year-old primigravida was referred at 24 weeks gestation for prenatal echocardiography. The echocardiographic findings were consistent with a prenatal ultrasound diagnosis of tetralogy of Fallot (TOF). After detailed counseling, the couple decided to terminate the pregnancy and undergo genetic testing. A trio (fetus and the parents) whole-exome sequencing (WES) and copy number variation sequencing (CNV-seq) were performed. CNV-seq identified no chromosomal abnormalities. WES analysis revealed a pathogenic, de novo heterozygous frameshift variant in *SMARCC2* (NM_003075.5: c.3561del, p.Leu1188fs). The genetic diagnosis of CSS8 was considered given the identification of the *SMARCC2* pathogenic variant.

**Conclusions:**

We report the first prenatal case with the *SMARCC2* variant. The presence of CHD further broadens the phenotypic spectrum of *SMARCC2*-related disease.

## Background

Coffin-Siris syndrome-8 (CSS8; OMIM: 618362) is a newly recognized syndromic neurodevelopment disorder characterized by variable degrees of impaired intellectual development and dysmorphic features [1]. It is a rare, autosomal dominant disorder caused by pathogenic variants in *SMARCC2* (OMIM: 601734). *SMARCC2* is one of the invariable core subunits of the chromatin-remodeling complex BRG1-associated factor (BAF), which plays a crucial role in embryogenesis and cardiac development [2]. To date, only 13 unique *SMARCC2* pathogenic variants, identified among 15 variably affected individuals, have been reported, two of which had cardiovascular abnormalities including left coronary distension and mild aortic dilatation [1]. Despite evidence that *SMARCC2* is essential for cardiac differentiation and driving stage-specific cardiac gene expression programs [2], none of the individuals with *SMARCC2* variants have been associated with congenital heart disease (CHD). At the same time, pathogenic variants in genes encoding other components of the BAF complex have been associated with CHD [3]. In fact, due to the scarcity of clinical reports, the clinical characteristics of CSS8, especially the low penetrant phenotype, such as cardiovascular abnormalities, have not been fully determined. We provide herein the first prenatal case with CHD that may be part of this newly identified *SMARCC2*-related disorder, which could help define its phenotypic spectrum and diagnostic criteria.

## Case presentation

A 28-year-old pregnant woman was referred to our center for prenatal echocardiography at 24 weeks of gestation with fetal cardiac abnormalities identified during routine second-trimester screening. The woman was in good health, with no family history of cardiovascular disease and no medication history during pregnancy. She and her husband are not consanguineous.

### Clinical phenotypes

**Fig. 1:**
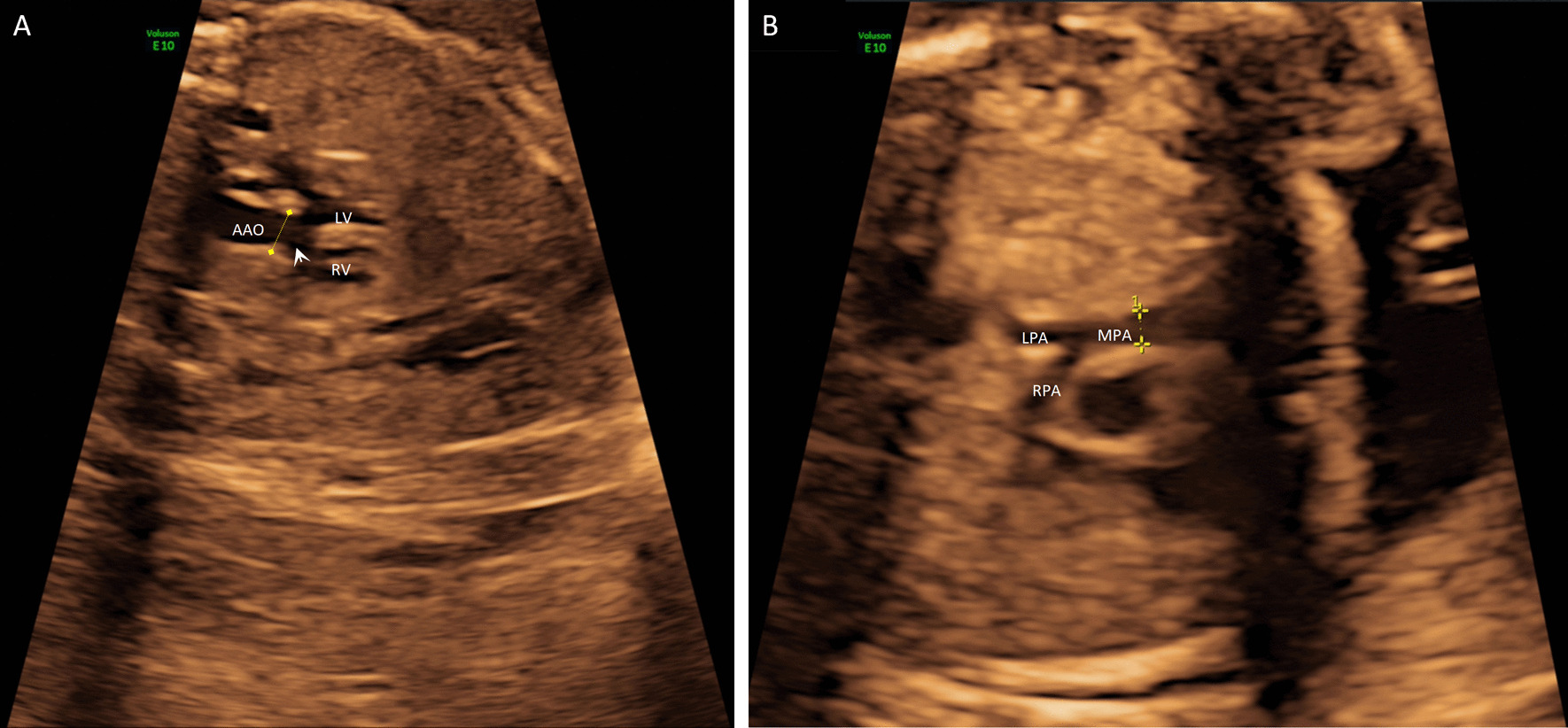
Cardiac defects in the fetus. **A** The dotted line indicates an enlarged aortic valve; the arrow indicates the ventricular septal defect. **B** The dotted line refers to a narrowing of the right ventricle outflow tract and pulmonary stenosis. AAO, ascending aorta; L/RPA, left/right pulmonary artery; LV, left ventricle; MPA, main pulmonary artery; RV, right ventricle

Detailed fetal echocardiographic examination revealed a subaortic ventricular septal defect (3.4 mm) with an enlarged aortic valve (Fig. 1A) and a right ventricular outflow tract and pulmonary stenosis (Fig. 1B). The echocardiographic findings supported the diagnosis of tetralogy of Fallot. After a detailed consultation, the couple decided to terminate the pregnancy and undergo genomic sequencing but declined an autopsy.

### Molecular findings

**Fig. 2:**
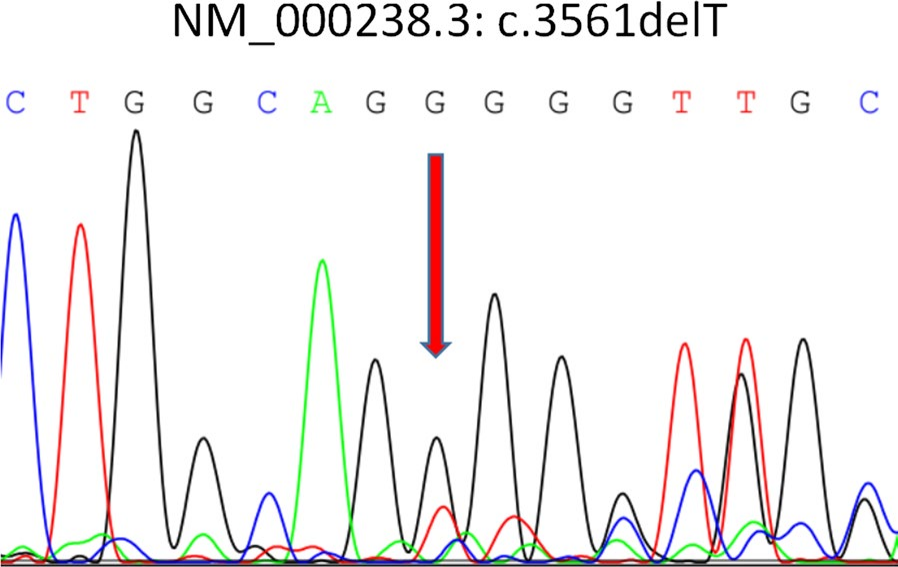
Sanger sequencing shows a frameshift variant in the fetus

We performed a trio (fetus and the parents) whole-exome sequencing and copy number variation sequencing using methods described previously [4, 5]. We found no chromosomal abnormalities or disease-causing variants in the known CHD genes. However, we identified a de novo heterozygous frameshift variant in *SMARCC2* (NM_003075.5: c.3561del, p.Leu1188fs) in the fetus (Fig. 2). This variant has not yet been described as pathogenic or benign and has not been reported in the general population database (gnomAD: https://gnomad.broadinstitute.org). It is predicted to result in either an abnormal truncated protein product or loss of protein from this allele via nonsense-mediated mRNA decay. Furthermore, recent evidence indicates that *SMARCC2* is intolerant to loss-of-function variants [1]. In conclusion, we classified this variant as pathogenic according to the American College of Medical Genetics and Genomics guidelines [6].

### Literature review

Since none of the previously reported patients [1] had CHD, the association between *SMARCC2* variants and CHD is uncertain. We further conducted literature and public database surveys to determine the association between SMARCC2 variants and CHD. We identified three additional patients with congenital heart disease, two of which carried a contiguous gene deletion containing *SMARCC2*, and one had a de novo *SMARCC2* loss-of-function variant [7, 8]. Detailed genotypes and phenotypes of these patients are summarized in Table 1.

**Table 1:** Clinical phenotype and genotype of four unrelated patients with congenital heart disease carrying *SMARCC2* deletion or loss-of-function variant

ID	Sex	Age	Cardiac phenotype	Extra cardiac phenotype	Genetic abnormality	Origin	Reference
401,720	Female	Infancy	Ventricular septal defect	Broad foot, broad palm, delayed speech and language development, feeding difficulties in infancy, hypertelorism, intellectual disability, muscular hypotonia, open mouth, premature birth, Strabismus, Thick eyebrow, Thick lower lip vermilion, Thick upper lip vermilion,Wide mouth	[hg19]del(12)(q13.3q14.2p22.3) chr12:g.56554154_63870277del	De novo	DECIPHER database*
F6	Female	Fetus	Cardiac malposition of the great arteries and multiple ventricular septal defects	Abdominal situs inversus	*SMARCC2* (ENST00000267064: c.1555C > T, p.His519Ter) and *NF1*(ENST00000456735:c.2747G > A,p.His916Gln)	De novo	Carss et al. (2014)
None	Female	22 years old	Congenital perimebranous ventricular defect	Neonatal respiratory distress syndrome,neurodevelopmental delay, poor verbal language, dysmorphic facial features, skeletal abnormalities, trigeminal nerve palsy, bilateral mixed hearing loss, rhinolalia, dysarthria and acquired dysphagia for solid foods	500 Kb-long deletion at 12q13.2-q13.3 that contains *SMARCC2* and other 25 genes	De novo	Roberti et al. (2018)
None	Female	Fetus	Tetralogy of Fallot	None	*SMARCC2* (NM_003075.5: c.3561del, p.Leu1188fs)	De novo	This study

*https://decipher.sanger.ac.uk/

## Discussion and conclusions

We report the first prenatal case of congenital heart disease with a novel variant in *SMARCC2*, a core subunit of the BAF complex. The discovery of pathogenic variants for pregnancies with structural anomalies during the prenatal period is essential for establishing a precise diagnosis, treatment decision, and correct prognostication and for providing accurate genetic counseling for the perinatal decision-making [9]. Furthermore, our report enlarges the variant spectrum of *SMARCC2*, and suggests the potential association between CHD and *SMARCC2* pathogenic variants.

Growing evidence indicates subunits of the BAF complex are involved in diverse aspects of cardiac development, and the disruption of the BAF complex underlies the pathogenesis of CHD [2]. Actually, CHD is not uncommon in patients with variants in genes encoding BAF complex subunits [1, 3]. For example, in a recent review of patients with variants in genes encoding components of the BAF complex, the incidence of CHD in patients with *SMARCB1*, *SMARCA4*, *SMARCE1*, and *ARID1A* variants was 45%, 42%, 67%, and 38%, respectively, and the overall incidence was 44% [3]. As for *SMARCC2*, recent evidence indicates that it is essential for cardiac differentiation through facilitating cardiomyocyte differentiation and controlling temporal steps in cardiac differentiation [2], suggesting a potential general role for the BAF complex in CHD. *SMARCC2* variants were recently reported in 15 unrelated patients with impaired intellectual development with speech and behavioral abnormalities, hypotonia, and varying dysmorphism [1]. None of these patients had CHD, although two of them presented coronary distension or aortic dilatation.

To our knowledge, a total of four unrelated CHD patients with *SMARCC2* deletion or loss-of-function variants, including the fetus with TOF carrying the novel *SMARCC2* pathogenic variant described here, have been reported in the public database or literature [7, 8] (Table 1). The patient 401,720 in DECIPHER database [10] and the patient reported by Roberti et al. [7] carried contiguous gene deletions containing the *SMARCC2* gene. They both presented the major features of the CCS8, including intellectual disability, developmental delay with prominent speech delay, behavioral abnormalities, feeding difficulties at the neonatal period, hypotonia, and dysmorphic features, suggesting that the haploinsufficiency of *SMARCC2* may contribute significantly to their phenotypes. Interestingly, only the fetus presented herein had no other genetic abnormalities, but the other three patients had either single-gene variants or copy number deletions involving genes other than *SMARCC2*. It is hard to say whether abnormal SMARCC2 or other genetic abnormalities are the most likely cause of the three patients. The presence of CHD in four unrelated patients with *SMARCC2* deletion or loss-of-function variants suggests the possible causal association between *SMARCC2* variant and congenital heart disease. Still, it requires further genetic or functional confirmatory studies.

Notably, although we identified no obvious extracardiac malformations by ultrasound, it was still difficult to distinguish whether the congenital heart disease in this fetus was isolated or syndromic. First, subtle dysmorphic features cannot be determined by fetal ultrasound, and some phenotypes, particularly neurodevelopmental disorders, are impossible to identify in the prenatal setting [9]. Furthermore, because of the termination of pregnancy, it is impossible to know if it would have developed other developmental features of CSS8 in the future.

In conclusion, by combining our case and the evidence mentioned above, we conclude that CHD is an intrinsic phenotype of *SMARCC2*-related disease with reduced penetrance. Further investigations into the pathophysiological mechanism associated with *SMARCC2* variants and genotype–phenotype correlations are needed.

## Data Availability

The data that support the findings of this study are included in our article. The raw data of whole-exome sequencing of the family in this study are not publicly available to protect participant confidentiality. In addition, according to the People's Republic of China regulations on the management of human Genetic Resources, without approval, any unit or individual shall not disclose the original data of genetic resources involving clinical patients. However, the re-analysis of the whole-exome sequencing data are available from the corresponding author on reasonable request. If you want to request access to the data and re-analysis, please contact Prof. Yihua He (Email: heyihuaecho@hotmail.com) at Department of Echocardiography in Beijing Anzhen Hospital, Capital Medical University, Beijing, China.

## Abbreviations

BAF: BRG1-associated factor
CHD: Congenital heart disease
CSS8: Coffin-Siris syndrome-8

## References

[1] Machol K, Rousseau J, Ehresmann S, Garcia T, Nguyen T, Spillmann RC, Sullivan JA, Shashi V, Jiang YH, Stong N (2019). Expanding the spectrum of BAF-related disorders: de novo variants in SMARCC2 cause a syndrome with intellectual disability and developmental delay. Am J Hum Genet.

[2] Hota SK, Johnson JR, Verschueren E, Thomas R, Blotnick AM, Zhu Y, Sun X, Pennacchio LA, Krogan NJ, Bruneau BG (2019). Dynamic BAF chromatin remodeling complex subunit inclusion promotes temporally distinct gene expression programs in cardiogenesis. Development.

[3] Kosho T, Okamoto N (2014). Genotype-phenotype correlation of Coffin–Siris syndrome caused by mutations in SMARCB1, SMARCA4, SMARCE1, and ARID1A. Am J Med Genet C Semin Med Genet.

[4] Sun H, Hao X, Wang X, Zhou X, Zhang Y, Liu X, Han J, Gu X, Sun L, Zhao Y (2021). Genetics and Clinical Features of Noncompaction Cardiomyopathy in the Fetal Population. Front Cardiovasc Med.

[5] Sun H, Yi T, Hao X, Yan H, Wang J, Li Q, Gu X, Zhou X, Wang S, Wang X (2020). Contribution of single-gene defects to congenital cardiac left-sided lesions in the prenatal setting. Ultrasound Obst Gyn.

[6] Richards S, Aziz N, Bale S, Bick D, Das S, Gastier-Foster J, Grody WW, Hegde M, Lyon E, Spector E (2015). Standards and guidelines for the interpretation of sequence variants: a joint consensus recommendation of the American College of Medical Genetics and Genomics and the Association for Molecular Pathology. Genet Med.

[7] Roberti D, Conforti R, Giugliano T, Brogna B, Tartaglione I, Casale M, Piluso G, Perrotta S (2018). A novel 12q13.2–q13.3 microdeletion syndrome with combined features of diamond Blackfan Anemia, Pierre Robin sequence and Klippel Feil deformity. Front Genet.

[8] Carss KJ, Hillman SC, Parthiban V, McMullan DJ, Maher ER, Kilby MD, Hurles ME (2014). Exome sequencing improves genetic diagnosis of structural fetal abnormalities revealed by ultrasound. Hum Mol Genet.

[9] Best S, Wou K, Vora N, Van der Veyver IB, Wapner R, Chitty LS (2018). Promises, pitfalls and practicalities of prenatal whole exome sequencing. Prenat Diagn.

[10] Firth HV, Richards SM, Bevan AP, Clayton S, Corpas M, Rajan D, Vooren SV, Moreau Y, Pettett RM, Carter NP (2009). DECIPHER: Database of chromosomal imbalance and phenotype in humans using ensembl resources. Am J Hum Genet.

